# Intensity modulated radiation therapy (IMRT): differences in target volumes and improvement in clinically relevant doses to small bowel in rectal carcinoma

**DOI:** 10.1186/1748-717X-6-63

**Published:** 2011-06-08

**Authors:** Henry Mok, Christopher H Crane, Matthew B Palmer, Tina M Briere, Sam Beddar, Marc E Delclos, Sunil Krishnan, Prajnan Das

**Affiliations:** 1 Department of Radiation Oncology, The University of Texas, M.D. Anderson Cancer Center, Houston, Texas, USA; 2 Department of Medical Dosimetry, The University of Texas, M.D. Anderson Cancer Center, Houston, Texas, USA; 3 Department of Radiation Physics, The University of Texas, M.D. Anderson Cancer Center, Houston, Texas, USA

## Abstract

**Background:**

A strong dose-volume relationship exists between the amount of small bowel receiving low- to intermediate-doses of radiation and the rates of acute, severe gastrointestinal toxicity, principally diarrhea. There is considerable interest in the application of highly conformal treatment approaches, such as intensity-modulated radiation therapy (IMRT), to reduce dose to adjacent organs-at-risk in the treatment of carcinoma of the rectum. Therefore, we performed a comprehensive dosimetric evaluation of IMRT compared to 3-dimensional conformal radiation therapy (3DCRT) in standard, preoperative treatment for rectal cancer.

**Methods:**

Using RTOG consensus anorectal contouring guidelines, treatment volumes were generated for ten patients treated preoperatively at our institution for rectal carcinoma, with IMRT plans compared to plans derived from classic anatomic landmarks, as well as 3DCRT plans treating the RTOG consensus volume. The patients were all T3, were node-negative (N = 1) or node-positive (N = 9), and were planned to a total dose of 45-Gy. Pairwise comparisons were made between IMRT and 3DCRT plans with respect to dose-volume histogram parameters.

**Results:**

IMRT plans had superior PTV coverage, dose homogeneity, and conformality in treatment of the gross disease and at-risk nodal volume, in comparison to 3DCRT. Additionally, in comparison to the 3DCRT plans, IMRT achieved a concomitant reduction in doses to the bowel (small bowel mean dose: 18.6-Gy IMRT versus 25.2-Gy 3DCRT; p = 0.005), bladder (V_40Gy_: 56.8% IMRT versus 75.4% 3DCRT; p = 0.005), pelvic bones (V_40Gy_: 47.0% IMRT versus 56.9% 3DCRT; p = 0.005), and femoral heads (V_40Gy_: 3.4% IMRT versus 9.1% 3DCRT; p = 0.005), with an improvement in absolute volumes of small bowel receiving dose levels known to induce clinically-relevant acute toxicity (small bowel V_15Gy_: 138-cc IMRT versus 157-cc 3DCRT; p = 0.005). We found that the IMRT treatment volumes were typically larger than that covered by classic bony landmark-derived fields, without incurring penalty with respect to adjacent organs-at-risk.

**Conclusions:**

For rectal carcinoma, IMRT, compared to 3DCRT, yielded plans superior with respect to target coverage, homogeneity, and conformality, while lowering dose to adjacent organs-at-risk. This is achieved despite treating larger volumes, raising the possibility of a clinically-relevant improvement in the therapeutic ratio through the use of IMRT with a belly-board apparatus.

## Background

Although surgery is necessary to achieve long-term cure for locally-advanced rectal cancer, randomized data has demonstrated the role for adjuvant therapy in this disease. The use of adjuvant radiation has been shown to significantly reduce the rate of local failure [1], with further improvement achieved with its concurrent administration with chemotherapy [2,3]. Moreover, Sauer and colleagues, demonstrated that preoperative chemoradiation was superior with respect to the rates of local recurrence and sphincter preservation compared to postoperative therapy [4]. The recently published NSABP R-03 trial demonstrated a significant improvement in 5-year disease-free survival with preoperative therapy, and a trend toward improved overall survival at 5-years [5].

The safe, effective, and tolerable administration of preoperative chemoradiation in rectal cancer is not without challenge, owing in part to the irradiation of a large volume at risk for microscopic disease spread, with potential toxicity to nearby bowel, bladder, and bones. Indeed, acute grade 3 or higher gastrointestinal toxicity in the form of severe diarrhea was reported to be 12% by Sauer and colleagues [4], with modern series reporting rates as high as 29%[6]. Additionally, a strong dose-volume relationship between the amount small bowel receiving intermediate- and low-doses of radiation and the rates of severe diarrhea has been demonstrated, particularly at the 15-Gy dose level [7-10]. Higher rates of acute severe toxicity may potentially lead to breaks in treatment or mitigate compliance, which may confer untoward consequences with respect to local control or survival [11].

Techniques have been utilized with the aim to reduce the volume of small bowel irradiated, such as the use of prone positioning with a belly-board apparatus to achieve bowel displacement away from the field [12]. Additionally, there has been interest in the application of highly conformal treatment approaches, such as intensity-modulated radiation therapy (IMRT). Whole-pelvis IMRT has been applied to gynecologic malignancy, with less toxicity than traditional 3D conformal radiation therapy (3DCRT)[13]. In anal cancer, IMRT has been compared to 3DCRT, showing similar target coverage with reduced dose to the genitals, femoral heads, small bowel, and iliac crest [14,15]. In comparison, the data for IMRT in rectal cancer are relatively sparse. Guerrero Urbano and colleagues compared IMRT with 3DCRT in five patients, and found small bowel sparing with IMRT only at the 40-Gy level and higher [16]. Tho and colleagues selected eight patients with the greatest volumes of small bowel irradiated from their cohort of patients, and observed an overall reduction in small bowel mean dose using IMRT, with evidence of sparing at high- and low- dose levels on a case-by-case basis [8]. In one of the largest series to date, Arbea and colleagues evaluated plans generated from 15 patients, and found using IMRT a significant reduction of dose to small bowel in the range of 40-Gy and higher; relationships at the intermediate- and low-dose levels were not explicitly reported [17]. Furthermore, the use of preoperative IMRT with concurrent capecitabine and oxaliplatin is currently under investigation in the recently completed phase II protocol, RTOG 0822 [18].

Therefore, the aim of our study is to further elucidate the potential role for IMRT in the management of locally-advanced carcinoma of the rectum with respect to minimizing dose to relevant normal tissue structures including the bladder, bones, and bowel, through direct dosimetric comparisons with 3DCRT techniques.

## Methods

### Patients

Ten patients recently treated preoperatively for adenocarcinoma of the rectum at the University of Texas M.D. Anderson Cancer Center were identified. These patients were representative of the breadth of disease typically encountered at this institution for preoperative chemoradiotherapy. Six patients were male, and four were female. All ten patients had clinical T3 disease. One patient was clinically node-negative, while nine were clinically node-positive. No patient had evidence of distant metastasis. All patients received concurrent fluoropyrimidine-based chemotherapy, typically with capecitabine.

### 3-field belly board plans

All patients were simulated and received treatment in the prone position using a carbon-fiber belly board apparatus (CIVCO Medical Systems, #125012) to achieve displacement of abdominal contents, which is the current standard practice at our institution. Computed tomography (CT) simulation was used in all patients. No specific bladder filling instructions were given to patients. No bowel contrast agent was used at the time of simulation. The plans used clinically [henceforth: 3-field belly board (3FBB)] consisted of a primary treatment to a prescribed dose of 45-Gy using a 3-field approach (PA and opposed laterals with wedges), typically without the use of any field-in-field optimization, followed by a localized boost for an additional 5.4-Gy using opposed lateral fields, using exclusively 18-MV photons and 1.8-Gy daily fractions. The intended targeted tissues included the gross tumor and nodal disease, which were contoured based on the CT simulation scan, mesorectum, and the internal iliac and presacral lymph nodes. Classic anatomical field borders were employed, with the superior field border at L5/S1, and inferior border at the level of the ischial tuberosities or 3-cm below the caudal-most extent of the tumor. For the PA field, the lateral field borders were placed 2-cm beyond the pelvic inlet. For the lateral fields, the anterior border was 3-cm anterior to the sacral promontory, and the posterior border was placed sufficient to expose a 1-cm margin on the posterior sacral bony contour. Multileaf collimator (MLC) blocking was utilized to block normal tissues outside of the intended targeted tissues. For the purposes of this study, given a lack of consensus with regard to delineation of boost volumes for rectal cancer [19], only the 45-Gy primary fields were evaluated.

### Target volumes and dose prescription for 3DCRT and IMRT planning

An IMRT plan as well as a 3DCRT plan designed to cover the PTV (henceforth: *3DCRT*) were generated for each patient from the initial CT simulation scan data. All cases were contoured by a single physician, and subsequently reviewed by an attending physician. Delineation of the clinical target volume (CTV) included the gross tumor and involved lymph nodes, mesorectum, presacral and internal iliac lymph node regions, with appropriate margin, as described in the RTOG consensus contouring atlas for anorectal cancer [19]. CTV to planning target volume (PTV) expansions of 7-mm were applied.

As noted above, the total prescription dose used in this study was limited to 45-Gy in 1.8-Gy daily fractions, without further boost.

### Organs at risk (OAR)

The relevant OAR volumes for this study were the bladder, femoral heads/necks, pelvic bones, small bowel, sigmoid/colon, and normal tissues. The bladder was contoured according to the CT simulation scan. The femoral heads/necks contours consisted of the bilateral femoral heads and necks to the level of the lesser trochanter. The pelvic bones contours were defined as the exterior of the bony table from top of the iliac crests to the ischial tuberosities. Differentiation of small bowel from sigmoid and colon was aided through correlation with the diagnostic, contrast-enhanced CT study closest in time to the date of simulation. The small bowel and sigmoid/colon volumes consisted of individual loops of bowel, contoured up to 2-cm above the superior-most PTV slice. The normal tissues contours were defined by the external contour, extending to 2-cm above and below the superior- and inferior-most PTV slices, respectively.

### Radiotherapy planning

All plans were generated using the Pinnacle version 8.0 m treatment planning system (Philips Healthcare), using MLC-equipped megavoltage linear accelerator delivery. For the 3DCRT and IMRT plans, the original CT simulation datasets from each patient were restored, and contoured as delineated above. For the 3DCRT plans, the field borders were modified from the 3FBB plans with the goal of covering greater than 95% of the PTV volume with the prescription dose, which was prescribed to the isocenter or a calculation point, and renormalized based on PTV coverage. Additional field-in-fields were utilized in all cases for homogeneity control, to limit hotspots to 107% of the prescription dose, particularly to anterior, bowel-containing regions. 18-MV photons were used for all 3DCRT plans.

IMRT treatment plans were generated with respect to delivery using only 6-MV photons via linear accelerators equipped with Millennium 120 MLC (Varian Medical Systems). Several beam arrangements were tested, with optimal results achieved using a 7-beam arrangement with the following gantry angles: 0°, 40°, 70°, 95°, 265°, 290°, and 320°. The collimator was set to 90°, with a total of 70 control points allocated to all beams. Direct machine parameter optimization (DMPO) was used, and at the discretion of the optimization algorithm, fields were split for all beam angles. In terms of general planning strategy, highest priority was given to PTV coverage, then to minimizing dose to small bowel. Of intermediate priority were reducing dose to the pelvic bones, bladder, and normal tissues outside the contoured regions; no specific optimization for sigmoid/colon volume was performed, but instead a general anterior abdominal contents avoidance structure was used. Lowest effort was applied to minimizing dose to the femoral head/neck. Collapsed-cone (CC) convolution methods were employed for final dose calculations. The final IMRT plans were independently reviewed and deemed clinically acceptable by both a gastrointestinal clinical physicist and radiation oncologist.

### Plan evaluation and statistical tools

Evaluated volumes included the PTV and relevant normal tissue volumes. The PTV, bladder, pelvic bones, femoral heads/necks, and small bowel were reported as whole volumes. The sigmoid/colon and normal tissue were reported exclusive of any overlapping/encompassed PTV.

Dosimetric parameters were calculated using tabular cumulative dose volume histogram (DVH) data, set to a bin size of 1-cGy, with median values reported. By convention, D_X% _= dose received by X% of the volume of interest, and V_X Gy _= percent volume of interest receiving at least a dose of X Gy. Maximum dose was expressed as D_1%_, minimum dose as D_99%_, mean dose as D_mean_, and maximum point dose as D_max_. The homogeneity index (HI) and conformality index (CI) were calculated for the 3DCRT and IMRT plans. HI was expressed as (D_5% _- D_95%_) / prescription dose. CI was expressed as the ratio of the absolute volume receiving the prescription dose to the volume of the target, V_45Gy _/ V_PTV_.

Plan average cumulative DVH values were calculated by exporting tabular DVH data set to a bin size of 10-cGy, and were plotted. For the small bowel, a curve based on the absolute volume irradiated was also generated. Integral dose to all tissues (including PTV) was calculated from the differential DVH data set to 10-cGy bin size.

For statistical analysis, each patient's IMRT plan was compared in a pairwise manner with both the 3FBB and 3DCRT plans, respectively. Non-parametric statistical analyses were performed using the paired, two-tailed Wilcoxon signed-rank test, with *p*-value < 0.05 taken to be significant.

## Results

### Dose to target volumes

When comparing the 3FBB treatment volumes to the contoured volumes based on RTOG consensus guidelines, it was evident that the contoured PTV encompassed a typically larger volume than that treated in the 3FBB plans. This was most pronounced superiorly, but was also seen in the extent of the PTV anterior to the sacral promontory, and occasionally in the inferior extent of the field. Indeed, dosimetric comparisons between 3FBB and IMRT plans, as shown in Table 1, revealed that the percentage of the PTV receiving the prescription dose was significantly lower for the 3FBB plans than with IMRT (V_45Gy_: median 3FBB 87.2% versus IMRT 99.5%; p = 0.005). Therefore, a 3DCRT plan was generated in each case using techniques described in the methods to adequately cover the PTV. This was quite effective, as the 3DCRT V_45Gy _was increased to a median of 98.4%, though still statistically inferior compared with IMRT (p = 0.02). Mean doses were similar between the 3DCRT and IMRT plans (p = 0.46).

**Table 1:** Dosimetric comparison of IMRT with 3DCRT: median value (range)

Volume	Parameter	IMRT	3FBB	3DCRT
PTV	D_mean _(Gy)	46.6 (46.4 - 46.9)	46.0 (45.6 - 47.0)*	46.6 (46.3 - 48.3)
1547 cm^3^	V_45Gy_	99.5% (98.7% - 99.8%)	87.2% (80.0% - 93.4%)**†**	98.4% (97.7% - 99.6%)*
(1459 -	D_99% _(Gy)	45.3 (44.8 - 45.8)	35.2 (10.7 - 40.3)**†**	44.9 (44.2 - 45.2)*
1968 cm^3^)	D_1% _(Gy)	47.6 (47.2 - 47.9)	48.3 (47.7 - 50.5)**†**	47.5 (47.1 - 48.2)
	HI	3.2% (2.5% - 3.6%)	N/A	4.2% (3.0% - 5.3%)**†**
	CI	1.16 (1.09 - 1.23)	N/A	1.35 (1.27 - 1.38)**†**
Bladder	D_mean _(Gy)	38.6 (31.1 - 42.4)	37.9 (27.5 - 44.2)	41.8 (31.0 - 45.0)**†**
72 cm^3^	V_30Gy_	74.7% (40.8% - 90.0%)	72.6% (36.7% -96.6%)	85.8% (47.2% - 100%)**†**
(32-652 cm^3^)	V_40Gy_	56.8% (26.2% - 76.6%)	58.5% (27.6% - 84.0%)	75.4% (38.0% - 100%)**†**
Femoral heads	D_mean _(Gy)	27.1 (20.8 - 29.6)	24.9 (22.4 - 30.7)	28.5 (21.9 - 31.8)*
211 cm^3^	V_30Gy_	28.0% (17.8% - 44.2%)	22.6% (12.0% - 33.3%)	31.9% (13.2% - 57.4%)
(151-393 cm^3^)	V_40Gy_	3.4% (1.1% - 7.0%)	6.3% (1.9% - 13.2%)*	9.1% (3.5% - 14.6%)**†**
Pelvic bones	D_mean _(Gy)	34.2 (30.5 - 36.2)	34.7 (31.9 - 36.8)*	36.7 (32.3 - 38.4)**†**
914 cm^3^	V_30Gy_	69.8% (55.6% - 76.3%)	66.7% (61.8% - 72.3%)	74.9% (63.4% - 81.0%)
(725-1338 cm^3^)	V_40Gy_	47.0% (35.2% - 52.8%)	53.9% (46.5% - 59.2%)**†**	56.9% (41.3% - 63.6%)**†**
Sigmoid/Colon	D_mean _(Gy)	18.9 (10.4 - 27.9)	17.5 (9.8 - 23.6)	25.5 (13.7 - 31.1)**†**
outside PTV	V_20Gy_	41.6% (13.2% - 72.6%)	38.0% (11.5% - 54.0%)	60.6% (24.9% - 75.2%)**†**
162 cm^3^	V_30Gy_	17.6% (5.1% - 48.1%)	10.4% (3.0% - 36.9%)	36.9% (10.8% - 63.2%)**†**
(23 - 389 cm^3^)	V_40Gy_	4.0% (0.7% - 19.2%)	2.4% (0.4% - 30.3%)	18.3% (5.1% - 38.5%)**†**
Small bowel	D_mean _(Gy)	18.6 (11.2 - 34.0)	21.0 (8.6 - 34.2)	25.2 (15.9 - 40.0)**†**
251 cm^3^	V_5Gy _(cc)	224 (2.5 - 526)	225 (2.2 - 525)	234 (2.5 - 530)
(3 - 537 cm^3^)	V_15Gy _(cc)	138 (0.1 - 257)	144 (0.4 - 413)*	157 (2.2 - 428)**†**
	V_25Gy _(cc)	81 (0.0 - 142)	79 (0.0 - 149)	123 (0.5 - 183)**†**
	V_40Gy _(cc)	45 (0.0 - 111)	50 (0.0 - 118)	76 (0.0 - 156)**†**
	V_45Gy _(cc)	37 (0.0 - 100)	33 (0.0 - 74)	53 (0.0 - 121)**†**
Normal tissues	D_mean _(Gy)	19.5 (12.0 - 21.6)	17.5 (12.8 - 20.6)*	20.5 (17.4 - 22.4)**†**
10.3*10^3 ^cm^3^	V_10Gy_	69.5% (58.0% - 76.9%)	64.9% (53.3% - 75.6%)*	73.8% (62.2% - 80.7%)**†**
(7.7*10^3 ^-	V_20Gy_	48.3% (39.5% - 52.7%)	42.0% (32.6% - 52.7%)*	50.5% (40.0% - 58.3%)*
18.9*10^3 ^cm^3^)	V_30Gy_	20.3% (16.5% - 27.4%)	17.3% (12.6% - 20.2%)*	23.5% (16.8% - 27.8%)
	V_40Gy_	6.7% (4.0% - 9.2%)	8.3% (4.2% - 10.9%)*	11.0% (6.7% - 15.7%)**†**
	V_45Gy_	2.3% (1.2% - 5.1%)	4.4% (2.1% - 6.0%)**†**	5.2% (2.8% - 6.4%)**†**

*Abbreviations*: PTV = planning target volume; IMRT = intensity modulated radiation therapy; 3FBB = 3 field belly board; 3DCRT = 3 dimensional conformal radiation therapy; HI = homogeneity index; CI = conformality index; for definitions of dosimetric parameters, refer to text; denotes statistically significant difference with IMRT as comparator, ***p ***< 0.05 (*) or ***p ***< 0.01 (**†**); otherwise, not statistically significant.

**Figure 1:**
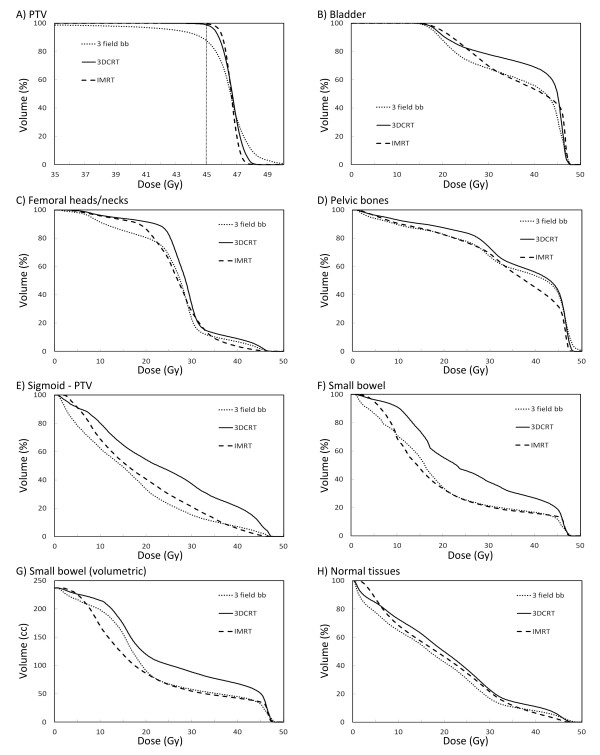
**Averaged cumulative dose-volume histograms**. Averaged cumulative dose-volume histograms for (A) PTV, (B) bladder, (C) femoral heads and necks, (D) pelvic bones, (E) sigmoid outside of PTV, (F) small bowel (relative), (G) small bowel (volumetric), and (H) normal tissues outside PTV, for IMRT, 3FBB, and 3DCRT.

With respect to target coverage, the minimum dose to the PTV, D_99%_, was higher with IMRT compared to the 3FBB (p = 0.005) and 3DCRT (p = 0.01) plans. Maximum dose to the PTV, D_1%_, was significantly lower with IMRT in comparison to 3FBB (p = 0.007); results were similar between IMRT and 3DCRT (p = 0.35). Both the homogeneity and conformality indices were significantly better with IMRT compared to 3DCRT (p = 0.007 and p = 0.005, respectively). Graphically, these findings are reflected in the averaged cumulative DVH plot (Figure 1A).

### Dose to organs at risk and normal tissues

With respect to mean dose, IMRT compared to 3FBB showed little difference for the bladder, femoral heads, sigmoid, and small bowel. However, compared to 3DCRT, IMRT resulted in significantly lower mean dose to the bladder (p = 0.007), sigmoid (p = 0.005), small bowel (p = 0.005), and to the femoral heads (p = 0.03). Mean dose to the pelvic bones was significantly lower with IMRT compared with either 3FBB (p = 0.04) or 3DCRT (p = 0.005).

With respect to high dose, IMRT significantly improved the V_40Gy _to the femoral heads (p = 0.01) and pelvic bones (p = 0.005) compared to 3FBB, and to the bladder (p = 0.005), femoral heads (p = 0.005), and pelvic bones (p = 0.005) in comparison to 3DCRT. For the dose to sigmoid/colon, IMRT was comparable to 3FBB at all dose levels evaluated, but was significantly lower compared to 3DCRT (p = 0.005).

Volumetric evaluation of total small bowel was performed at dose levels ranging from 5- to 45-Gy. When IMRT was compared to 3FBB, the V_15Gy _was significantly reduced with IMRT (p = 0.03), but similar at other doses. IMRT compared to 3DCRT showed significant reductions in the volumes of small bowel irradiated at levels ranging from 15- to 45-Gy (p < 0.01). With respect to V_15Gy_, the magnitude of the difference in median volumes was modest (138-cc IMRT versus 157-cc 3DCRT; p = 0.005) when evaluating the ten patients as a whole. However, the most profound bowel sparing was evident in the subset of patients with the largest volume of small bowel in proximity to the treatment field. For example, in the 6 patients with the highest volume of small bowel (range: 209 - 537-cc), the volume of bowel receiving 15-Gy was reduced from a median of 231-cc in the 3DCRT plans to 185-cc with IMRT. Conversely, in the remaining four patients, only a slight absolute reduction was evident (median V_15Gy_: 13-cc IMRT versus 22-cc 3DCRT).

Normal tissues outside the target were evaluated, and IMRT plans had a significantly higher mean dose (p = 0.02) and V_10Gy _(p = 0.01) to V_30Gy _(p < 0.02) in comparison to the 3FBB plans. However, at the highest doses, IMRT was significantly lower (V_40Gy_, p = 0.02; V_45Gy_, p < 0.01). IMRT, compared to 3DCRT, had a significantly lower mean dose (p = 0.007), V_40Gy _(p = 0.005) and V_45Gy _(p = 0.005), with more modest, but significant, differences at V_10Gy _(p = 0.005) and V_20Gy _(p = 0.01).

**Figure 2:**
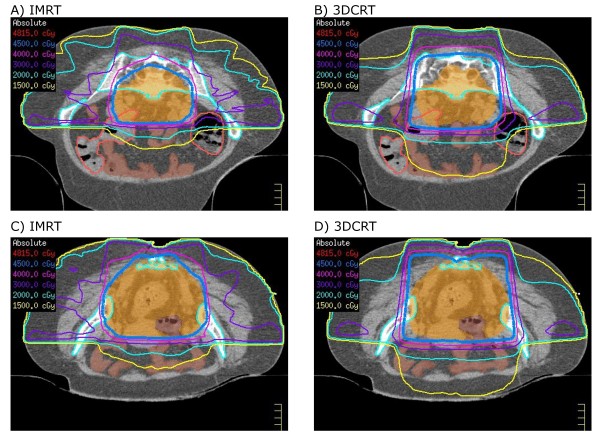
**Representative axial slices**. Representative axial slices showing isodose distributions for two planes for an (A), (C) IMRT and (B), (D) 3DCRT plan.

Averaged cumulative DVH plots for organs-at-risk and normal tissues are depicted in Figure 1. Representative axial slices showing isodose distributions for an IMRT and a 3DCRT plan for one patient are shown in Figure 2.

### Plan summary characteristics

Monitor units were significantly higher with IMRT compared to either 3FBB (p = 0.005) or 3DCRT (p = 0.005) (Table 2). The overall plan maximum doses were similar between IMRT and 3FBB, but higher with IMRT compared to 3DCRT (p = 0.005). Integral dose, calculated for all tissues including the target volume, was significantly higher for IMRT compared to 3FBB (p = 0.007), but lower compared to 3DCRT (p = 0.007).

**Table 2:** Plan summary comparison of IMRT and 3DCRT plans: median value (range)

Parameter	IMRT	3FBB	3DCRT
MU/fraction	786 (730 - 950)	238 (224 - 272)**†**	242 (232 - 276)**†**
D_max _(Gy)	48.8 (48.4 - 49.4)	48.8 (48.1 - 51.0)	48.2 (47.8 - 49.2)†
Integral dose	2.74 (2.39 - 4.03)	2.56 (2.15 - 3.60)**†**	2.86 (2.49 - 4.12)†
(Gy*cm^3^*10^-5^)			

*Abbreviations*: MU = monitor units; IMRT = intensity modulated radiation therapy; 3FBB = 3 field belly board; 3DCRT = 3 dimensional conformal radiation therapy;denotes statistically significant difference with IMRT as comparator, ***p ***< 0.05 (*) or ***p ***< 0.01 (**†**); otherwise, not statistically significant.

## Discussion

In this study, we found that the application of IMRT for rectal cancer gave excellent results in comparison to non-IMRT based approaches. With respect to the PTV, we found that IMRT plans achieved superior coverage, homogeneity, and conformality in treating the gross disease and at-risk pelvic nodal volume, in comparison to 3DCRT plans targeting the PTV. This was not at the expense of adjacent organs-at-risk, as some measure of sparing was evident for all organs-at-risk evaluated: small bowel, sigmoid, pelvic bones, bladder, and femoral heads (IMRT versus 3DCRT). In this comparison, IMRT actually decreased the overall integral dose to all tissues, and achieved lower mean doses to normal tissues outside the PTV, which was evident especially in the high dose range. As expected, IMRT required significantly more monitor units per fraction, compared to 3DCRT.

We found quite interesting the discrepancy between the size of the volumes encompassed by the PTV, which were generated according to the RTOG consensus contouring atlas [19], and the volumes treated according to classic anatomic landmarks (3FBB), even considering the anticipated patient-to-patient anatomical variation. This was reflected in the significantly lower proportion of the PTV volume receiving the prescription dose in the 3FBB plans, and to a certain extent the significantly lower overall integral dose, compared to IMRT. We found that despite the significantly larger volume targeted in the IMRT plans, IMRT achieved either similar or improved dose levels to all organs-at-risk evaluated. For example, the small bowel irradiated had similar mean doses, and the absolute volumes irradiated were similar from the 5- to 45-Gy levels, except at 15-Gy, where IMRT was statistically improved, compared to the 3FBB plans.

In terms of acute, severe treatment-related toxicity, diarrhea is the most common, and studies have demonstrated a strong dose-volume relationship with small bowel irradiated [7-10]. Baglan and colleagues demonstrated a strong association between the rate of small bowel toxicity and the V_15Gy _level; when the V_15Gy _was below 150-cc, low rates of grade 2 or higher toxicity were observed, while the majority of patients with V_15Gy _over 300-cc had grade 3 or higher toxicity [7]. Subsequent studies by Robertson and colleagues have confirmed the significance of the V_15Gy _dose level, as well as other intermediate dose levels, including the V_20Gy _and V_25Gy_, with respect to severe diarrhea [9,10]. In our study, we found IMRT achieved significant sparing in terms of the mean dose to small bowel and absolute volumes from V_15Gy _to V_45Gy_, whereas no difference was seen at the lowest dose level evaluated, V_5Gy_, compared to the 3DCRT plans. This sparing at the V_15Gy _level was most pronounced in the cases with the highest volumes of small bowel within or nearby the PTV. Therefore, we would predict a lower rate of severe, acute gastrointestinal toxicity in these patients treated with IMRT. Furthermore, reduction in the small bowel V_45Gy _using IMRT may lead to lower rates of late gastrointestinal toxicity [20]. Again, in the comparison between IMRT and classic bony landmark-derived 3FBB fields, despite a more extensive volume treated with IMRT, we would predict similar, or based on the V_15Gy_, possibly improved rates of severe, acute gastrointestinal toxicity with IMRT compared to 3FBB.

In the context of other planning studies comparing IMRT with 3DCRT, we feel overall our results are superior and additive. Prior studies have demonstrated a reduction in small bowel mean dose [8], or improvement at the high-dose extreme [16,17], with the use of IMRT. With respect to positioning, while all three studies employed prone positioning, one achieved immobilization using a foam cushion [17], whereas two made no specific reference to the use of a bowel displacement device [8,16]. In contrast, using a rigid, carbon-fiber belly board apparatus, we observed a significant improvement in small bowel dose from 15-Gy through the 45-Gy level, as well as the mean dose, with IMRT compared to 3DCRT plans. Therefore, our study demonstrates a further significant interval improvement in small bowel dose is realized with the use of IMRT in conjunction with the carbon-fiber belly board. An additional strength of our study is that our contoured volumes conformed to the RTOG consensus guidelines.

We chose as a "class-solution" approach to use an asymmetric, seven-beam arrangement, biased against anterior-directed beams, thus minimizing beam entry through anterior-lying bowel contents or through the belly-board apparatus. This appeared to take advantage of strengths of the 3-field beam arrangement, namely sharp dose falloff in the intermediate- and low-dose range anteriorly. Indeed, recently-published studies of IMRT, using 5- to 9-equispaced beams, have principally demonstrated reduced small bowel mean dose and V_40Gy_, compared to 3DCRT [8,16,17]. In our study, in addition to these findings, we found IMRT capable of reducing small bowel volumes receiving potentially toxicity-inducing intermediate- and low-dose irradiation, at a statistically-significant level. Concomitantly, IMRT achieved superior PTV target coverage, homogeneity, and conformality, as well as evidence of sparing of all other organs-at-risk evaluated in this study. Again, our results support a clear dosimetric advantage for IMRT, even with the use of prone-positioning on a belly-board apparatus.

With respect to the volume of the irradiated target, there are at least two different ways to consider this issue. In our study, the PTVs, generated with a 7-mm expansion, were typically larger than the volume treated using classic 3FBB fields. Given the excellent historical results obtained with the classic 3FBB fields, one interpretation is that the target volumes, as delineated by the RTOG consensus IMRT contouring atlas for anorectal disease, may be more generous than necessary. Alternatively, as we found that the more comprehensive PTV target coverage was achieved without increasing dose to the organs-at-risk including the small bowel, it is conceivable that improved efficacy is attainable without increasing acute- and long-term toxicities through the use of IMRT. Long-term clinical data would be necessary to provide evidence for this. As an additional point, the use of IMRT does not automatically confer normal tissue sparing, as an excessively voluminous target volume may in fact lead to higher absolute volumes of normal tissues treated. This reinforces the importance of consensus target delineation to achieve standardization from practice-to-practice.

Due to daily setup uncertainties using the rigid carbon-fiber belly-board apparatus, for IMRT treatment of a CTV-to-PTV expansion of 7-mm used in this study, it may be worthwhile to consider daily kilovoltage imaging, or perhaps modifications such as the incorporation of a vacuum-cradle device to improve setup reproducibility.

One potential criticism for intensity modulated treatment approaches is with respect to integral dose, whereby larger volumes of normal tissues are exposed to lower radiation doses, which may lead to increased incidence of second malignancies [21]. In our study, we found a lower integral dose with IMRT compared to 3DCRT plans targeting the PTV. However, integral dose was slightly higher with IMRT than in the classic 3FBB plans.

Another potential downside of a static-field intensity modulated therapy approach is a longer beam-delivery time that is required as compared to 3DCRT, with respect to intrafractional motion. This may be overcome using volumetric-modulated arc therapy (VMAT) based techniques.

## Conclusions

For the adjuvant treatment of rectal carcinoma, IMRT, compared to 3DCRT, yielded plans superior with respect to target coverage, homogeneity, and conformality, while lowering dose to adjacent organs-at-risk. This benefit was seen additive to the use of prone-positioning on a belly-board apparatus, and with respect to small bowel toxicity, could potentially be clinically significant.

## Competing interests

The authors declare that they have no competing interests.

## Authors' contributions

HM carried out the study conception and design, drafted the manuscript, and performed treatment planning. PD carried out the study conception and design and drafted the manuscript. MBP performed treatment planning. TMB and SB performed physics checks/plan evaluation. Patient accrual and radiation field design were performed by CHC, MED, SK, and PD. CHC provided mentorship for this work. All authors read and approved the final manuscript.
